# Size Matters: Concurrency and the Epidemic Potential of HIV in Small Networks

**DOI:** 10.1371/journal.pone.0043048

**Published:** 2012-08-24

**Authors:** Nicole Bohme Carnegie, Martina Morris

**Affiliations:** 1 Department of Humanities and the Social Sciences in the Professions, New York University, New York, New York, United States of America; 2 Department of Statistics, University of Washington, Seattle, Washington, United States of America; 3 Department of Sociology, University of Washington, Seattle, Washington, United States of America; University of Cape Town, South Africa

## Abstract

**Background:**

Generalized heterosexual epidemics are responsible for the largest share of the global burden of HIV. These occur in populations that do not have high rates of partner acquisition, and research suggests that a pattern of fewer, but concurrent, partnerships may be the mechanism that provides the connectivity necessary for sustained transmission. We examine how network size affects the impact of concurrency on network connectivity.

**Methodology/Principal Findings:**

We use a stochastic network model to generate a sample of networks, varying the size of the network and the level of concurrency, and compare the largest components for each scenario to the asymptotic expected values. While the threshold for the growth of a giant component does not change, the transition is more gradual in the smaller networks. As a result, low levels of concurrency generate more connectivity in small networks.

**Conclusions/Significance:**

Generalized HIV epidemics are by definition those that spread to a larger fraction of the population, but the mechanism may rely in part on the dynamics of transmission in a set of linked small networks. Examples include rural populations in sub-Saharan Africa and segregated minority populations in the US, where the effective size of the sexual network may well be in the hundreds, rather than thousands. Connectivity emerges at lower levels of concurrency in smaller networks, but these networks can still be disconnected with small changes in behavior. Concurrency remains a strategic target for HIV combination prevention programs in this context.

## Introduction

The large, persistent disparities in HIV prevalence have stimulated much research. They are evident at every scale: from differences across global regions, with nearly 70% of all cases located in sub-Saharan Africa, to differences in geographically contiguous subpopulations, such as the nearly 10-fold difference in prevalence by race in the United States [1], [2], and persistent differentials of similar magnitude in countries like Kenya, Tanzania and South Africa [3], [4], [5].

Most heterosexual populations do not have generalized epidemics of HIV, but some do. So, what conditions are needed for a generalized epidemic of HIV to emerge? In contrast to air and water borne pathogens, HIV requires intimate personal contact to spread. The probability of such contact between any two individuals in a population is low, and after the acute phase of infection, the probability of transmission is low. Under these conditions the transmission network would typically lack the connectivity needed to sustain HIV transmission. Generalized epidemics would not be predicted.

This issue is often addressed in mathematical modeling by introducing extreme variation in the rates of partner acquisition. Core group models are one example of this [6], creating a small “high activity group” that can act as a reservoir for sustained transmission, and pass infection on to members of less active groups. Such models produce concentrated epidemics, however, with high prevalence in the core group, and a small trickle of infections out to the general population, where further transmission fails. In typical simulation models developed to reproduce the prevalence seen in generalized epidemics, the average male and female in the population is assumed to have 75–200 lifetime partners, and the top 6–12% of both sexes have 360–2,800 lifetime partners [7], [8], [9]. These partner assumptions are one or two orders of magnitude larger than any data observed in empirical studies of sexual behavior.

An alternative mechanism for creating connectivity without such extreme levels of heterogeneity is concurrency – partnerships that overlap in time [10], [11], [12]. With concurrency, the focus shifts to the *momentary* or cross-sectional distribution of the number of partners per person, rather than the cumulative distribution of partners over time. Serial monogamy produces a momentary distribution with only two values, 0 (no partners) and 1 (a single partner). Values above 1 in the momentary distribution indicate the presence of concurrency. It does not take a high rate of partner acquisition to generate connectivity in a network with concurrency. For example, imagine a population in which no one had more than 2 partners in their lifetime. If everyone has those partners concurrently, this creates a circle: everyone has only two partners, but the entire population is completely connected, and the network can rapidly spread infection. A serially monogamous network with only two partnerships per person over time would have far less transmission potential.

Concurrent partnerships become particularly important in the context of short infectivity windows [13]. In this situation, when the index case becomes infected by one partner, their concurrent partner is more likely to be contacted and exposed during the brief window period. There is no need for the index case to rapidly acquire new partners during this window. HIV causes a brief spike of infectivity immediately after acquisition [14], and simulation studies have shown that concurrency amplifies the impact of this acute spike [15], [16]. As this suggests, the primary impact of concurrency at the individual level is that it increases the probability of the index case transmitting infection [17], [18], [19].

**Figure 1:**
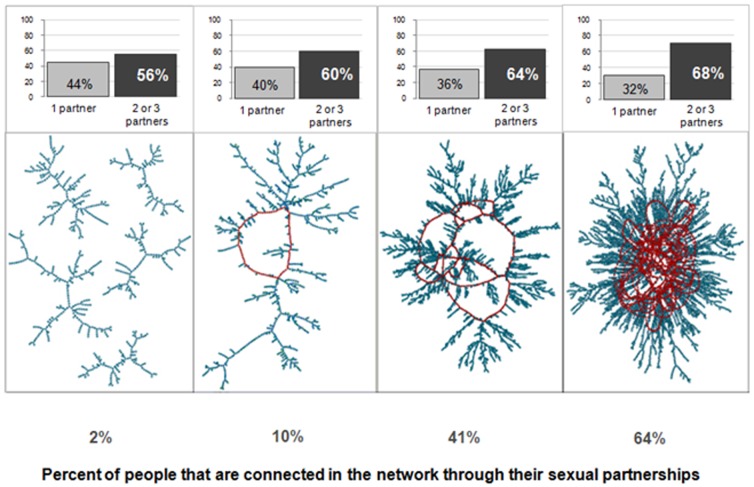
Growth of the largest component in large networks at the threshold: This figure demonstrates the rapid growth of the giant component in large networks near the threshold level of concurrency. The top row shows histograms of the momentary degree distribution for networks at mean degree 1.68, 1.74, 1.80 and 1.86, corresponding to 56, 60, 64 and 68 percent of sexually active persons having concurrent partners. The bottom row gives a visualization of a typical largest component. We can see that the percentage of the population in the largest component jumps rapidly from 2% to 64% with a very small increase in mean degree (0.2 of a partner, 12% more persons with concurrent partnerships).

The rapid and non-linear impact of concurrency on the connectivity of a network can be seen in Figure 1, which has been widely distributed to researchers, intervention program developers, and educators in the international HIV prevention community. The histograms at the top show the fraction of persons with concurrent partners (among those who have partners). No one in these simulated networks has more than three partners, and those with three partners are a small minority of partnered persons in all panels – 10% in the first panel and 18% in the last. The network visualizations in the lower panels show examples of the typical largest *connected component* for a 10,000 node network with that momentary degree distribution. Reading across the panels, the fraction of persons with concurrent partners rises by only 12%, and the *mean degree* (i.e., the average number of partners that sexually active people have at any moment in time) only increases by 0.2 of a partner, from 1.7 to 1.9, but the percentage of the population in the largest component jumps from 2% to 64%. This shows the highly non-linear nature of network connectivity: like infectious disease transmission and population growth it has a threshold, and the outcomes are qualitatively different above and below that threshold.

This paper was motivated by the reactions that this figure received during research study dissemination activities in a rural location in the Kisumu district of western Kenya. The dissemination involved the results of a longitudinal study of widow inheritance in the region, and it included an educational component on concurrency. After an interactive game used to demonstrate the way concurrency works to connect people, the participants were shown the graphic in Figure 1. When asked which panel of the Figure they thought was most similar to their local network, the vast majority of participants (virtually everyone) voted for panel 3. The original study was not designed to provide estimates of concurrency in this population, but it seemed unlikely to the researchers that the local momentary degree distribution had such high proportions of persons with two and three partners. At the same time, the near unanimity of the participants' perception, across different villages, seemed too strong a signal to ignore.

One way that the conflicting perceptions of researchers and villagers can be reconciled is if concurrency has an amplified effect in small networks. The simulations used to create Figure 1 were intentionally based on a large network. Network density declines with network size for any given mean degree, and the point of this Figure was to show that connectivity could be maintained, even for very large, very sparse networks with low mean degree. The study participants, however, live in villages that are much smaller than 10,000 persons. Table 1 shows the adult populations of 30 villages in four trial arms that are part of the next stage of research in this region. Village size ranges from about 100–400 persons. While residents may have some partners from outside the village, it is likely that the effective size of their sexual networks are much smaller than 10,000. Does that change the impact of concurrency on network connectivity?

**Table 1:** Adult populations of 30 villages in the Siyaya district of Kenya.

	Number of Adults in villages		
Region (number of villages)	Minimum	Maximum	Average
1. Kakumu/Kombewa (7)	129	359	240
2. Gangu (11)	89	187	144
3. North Rambula (5)	164	215	187
4. Masat (7)	163	281	220
All Villages	89	359	198

The data source is the Kenya National Bureau of Statistics 1999 Census.

## Materials and Methods

To answer this question, we simulated smaller networks across the range of degree distributions shown in Figure 1 and compared the average size of the largest component in these smaller networks to the size predicted by asymptotic analytical methods. We constrain sexual mixing to be heterosexual, and thus consider only bipartite networks.

### Simulation methods

We use a model-based simulation approach that produces a representative sample of networks with a specified degree distribution and size. Three sizes of networks were simulated that reflected the range of observed village sizes in Table 1: 100, 200 and 500 nodes. We divide the population evenly into males and females, and use the same degree distribution for both sexes. For each level of concurrency, we create a starting network with a stub-matching approach that produces a network of the desired size with a specific degree distribution by randomly permuting the edges emanating from females and pairing them with the list of edges emanating from males. The resulting list of edges is checked to for repeated (M,F) pairs, and re-permuted if any exist. We then sampled 2500 networks from the space of networks with this size and degree distribution using the *networksis* [20] package in the *statnet* program [20]. For each sampled network, we calculate the size of the largest component using the *sna* [21] package in *statnet*.

### Asymptotic Results

Newman [22] derived the size of the largest component in an infinite bipartite graph using probability generating functions (pgfs). The pgfs of the degree distributions for women and men are given by
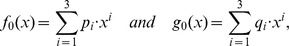
respectively. We also need the pgfs of the remaining degree of a node when an edge is randomly selected. These are
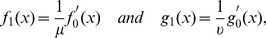



His results depend on two functions: 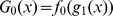, the number of female partners of a randomly selected woman's male partners, and 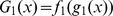, the number of female partners of the remaining male partners of the woman incident on a randomly selected edge. The threshold for the development of a giant component is 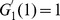. In this case, with equal degree distributions for men and women, and degree constrained to 1 to 3, this reduces to 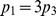, which yields a threshold of 1.72 for formation of a giant component. The percentage of women (and men) in the network who are in the giant component is given by 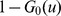, where *u* is the minimum positive solution of 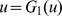.

## Results

**Figure 2:**
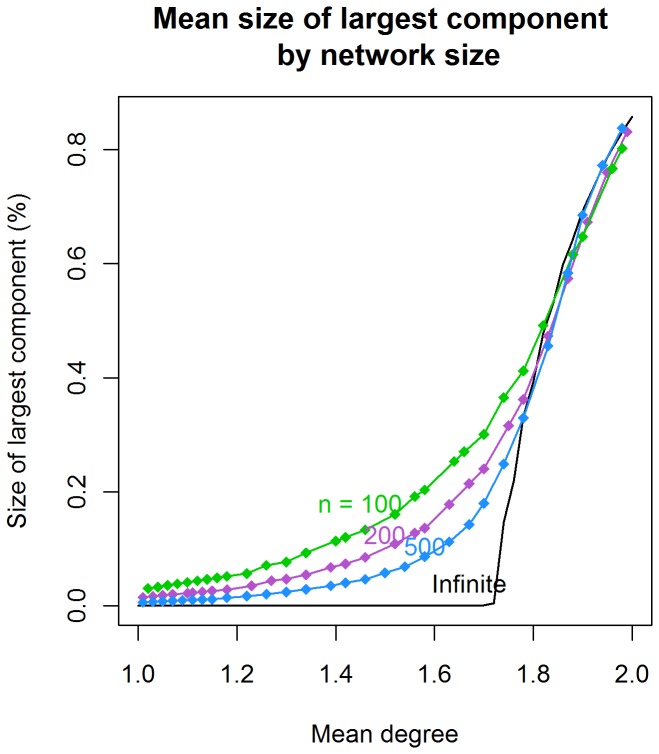
Mean size of the largest component as a function of network size and concurrency: We see here the average size of the largest component for networks of size 100, 200, and 500 nodes as the concurrency rises through the threshold level. The black line gives the analytic approximation for large networks. Notice that the curves for the small networks approach the analytic result as the network size increases. The small networks have the same threshold, but the behavior around the threshold is different. The flatter curve means that lower levels of concurrency produce higher levels of connectivity in small networks.

Figure 2 plots the average size of the largest component as a function of the momentary degree distributions for networks of size 100, 200 and 500, showing the corresponding analytic results for asymptotically large networks. Each point on the graph represents the mean of 2500 simulated networks for that scenario. The midpoint of the sharp increase in component size for the small networks occurs at the analytic threshold of 1.72. However, the transition across the threshold is more gradual. In particular, the smaller the network, the more rapidly connectivity rises before the analytic threshold is reached. For example, at a mean degree of 1.6 there is no large component in the asymptotically large networks, but a network with 100 persons would produce a large component connecting, on average, 20% of the population. The same personal behavior in the two networks would lead to different population level outcomes.

**Figure 3:**
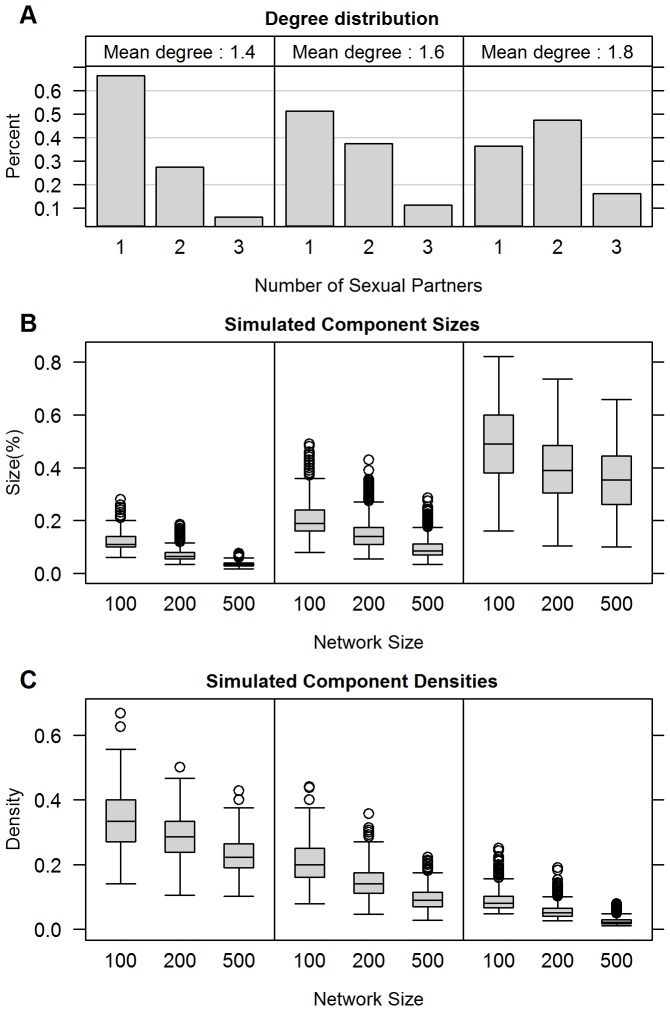
Distributions of the largest component size and density as a function of network size and concurrency: In this figure we reproduce some of the information in **Figure 1** for networks of size 100, 200 and 500. The top row again shows histograms of the momentary degree distribution for networks at mean degrees around 1.4, 1.6 and 1.8, corresponding to concurrency prevalence of 35, 50 and 65%. The middle row shows the size distribution of the largest component as a percentage of the network, and the bottom row shows the distribution of the density of the largest components. Note that the percentage of the network in the largest component decreases with network size, but variability rises with concurrency levels. The density of the largest component decreases with network size and level of concurrency.

Figure 3 replicates the information from the graphic in Figure 1 for the smaller networks of interest here. The top panel shows the histograms of the degree distribution for networks at mean degrees around 1.4, 1.6 and 1.8, and the bottom two panels show the corresponding distributions of the size and density of the largest component from simulated networks with these mean degrees. As in Figure 1, the connectivity in these networks arises without any highly active persons acting as hubs; no single node has a degree above 3 and over 80% have only one or two partners, even in the most highly connected graphs.

The inverse relation between component size and network size is readily apparent, but the variability in the size of the largest component also rises as both the mean degree and network size increase. As a result, the network size effects dominate when mean degree is low, but are moderated by variability as the mean degree rises.

The typical density of the largest component also decreases as network size increases, but it is inversely related to mean degree. This is somewhat counter-intuitive, as we might have expected that higher overall density would lead to higher density for the largest component. In fact, the opposite happens, because the component size is also increasing as mean degree rises, so the (n-1) nodes required to connect a component of size n quickly become a small percentage of the overall possible number of edges among n nodes. Results for bicomponents (not shown) are substantively similar.

## Discussion

This study has demonstrated that small networks amplify the effects of concurrency, producing high connectivity at partnership rates that are well below the asymptotic threshold for large component formation. This has a number of implications.

The first concerns the state of our knowledge regarding the size of a typical sexual network, and how much variation in size there is across different populations. We know very little empirically about these questions, but a number of factors suggest that there may be substantial variations in effective network size. One way such size differentials can emerge is in the context of a heterogeneous population with different sized subgroups and strong assortative mixing. In this context group size would be correlated to effective network size, and minority groups would have smaller networks.

Small networks may therefore emerge in the midst of large populations. In the United States, for example, empirical studies consistently show strong assortative mixing by race in sexual partnerships [23], [24]. In any particular geographic area, such assortative racial mixing may lead to fairly small effective networks for minority populations, despite the large populations in which they are embedded. The level of assortative mixing may be further constrained by high levels of residential segregation that isolate and concentrate different groups in the U.S. [25], [26]. As noted by Acevedo-Garcia [27], residential segregation can affect disease transmission both directly, by influencing the spatial distribution and contact patterns of populations, and indirectly, by concentrating risk factors such as poverty, overcrowding, lack of access to healthcare and social disorganization. Small networks are also likely to predominate in rural areas, where the geographic patchiness of population density reduces the pool of local partners and the effective size of the resulting network.

Questions remain, however, on how to define the boundaries and effective size of a network. There is evidence that these networks are not well described by a simple circular area around a person's residence [28]. While the majority of relationships may occur within a village, neighborhood or socially homogeneous group, and thus be constrained by the size of that unit, persons with concurrent partnerships may have one partner in their own group, and a second partner in another group. This is a common pattern associated with labor migration, for example, where the migrant has a partner both at home and at the work location [29]. This type of contact pattern – a network where within-group contacts predominate but there is the occasional contact out of the group – has received much attention in previous research in both epidemiology and social networks. Examples include the role of “long-jumps” for epidemic persistence in zoonotic diseases [30], the studies of diffusion in “small world” networks [31] in the social network literature, and the role of “bridge populations” for spreading infection to low risk populations in the HIV transmission literature [32]. Small world networks are one of the most efficient structures for diffusion. They maximize spread within groups, while preserving the connection between groups, using another form of heterogeneity to enable epidemic persistence in otherwise sparsely connected populations.

The second concerns the validity of mathematical modeling studies for HIV transmission analysis. Simulation methods like deterministic compartmental models that rely on asymptotic approximations or large population assumptions will fail to capture this element of size-dependent variation in network connectivity. Analytic methods for deriving standard qualitative measures of epidemic potential, e.g., R_0_ and doubling times also rely heavily on asymptotic approximations [33], [34], [35]. As a result, these methods may underestimate the role that concurrency has in explaining prevalence differentials across groups, and the contribution concurrency reduction can make to prevention.

The importance of population-specific combination prevention approaches to HIV has made mathematical modeling a necessary tool for planning efforts. Our findings suggest that it would be prudent to cross-validate large simulation studies with small scale simulations that use empirically accurate representations of the network size and structure. Our study focuses on the characteristics of static networks, but transmission occurs over dynamically evolving networks of relationships. All else equal, the higher connectivity we have observed in the cross section will translate into larger reachable paths in time. The impact of concurrency on the size of the reachable path in a dynamic network is not well understood, however, and this remains an important area for future research.

Finally, this has implications for empirical study design. The unique empirical signature of concurrency is a momentary distribution that is not constrained to 0 (isolates) and 1 (monogamously paired); this defines the point prevalence of concurrency. The impact of concurrency also depends on the intensity of the overlapping partnership intervals – determined largely by the duration of overlapping partnerships, and the level of activity in both partnerships during the overlap. As noted by the UNAIDS consensus paper, the minimal information needed to establish the point prevalence of concurrency and its intensity can be obtained from behavioral surveillance surveys by replacing the standard questions that elicit information on the cumulative distribution, e.g., “how many partners have you had in the last X months?” with partner-specific questions on the start and end dates of sexual relations, and whether the partnership is ongoing [36]. Our findings suggest that additional partner-specific information, e.g., age, race or geographic residence, would be helpful for identifying the clustering and segregation that can lead to small effective network size. Reducing the impact of social desirability bias in self-reported sexual behavior is a key part of improving the validity of the data.

This study highlights the potential importance of concurrency reduction as an element of comprehensive prevention, and the need for reliable empirical data on the prevalence and duration of concurrent partnerships. In large networks, the rapid changes in connectivity only occur near the threshold, which in this simplified example is when about 65% of the *partnered* population has concurrent partners. While this excludes single persons from the denominator, it is still a high level of concurrency. By contrast, in smaller networks, the connectivity effects occur across a wider range of concurrency levels, so changes at lower (and perhaps more realistic) levels of concurrency can still have an impact. For example, only 15% of the population would need to cut back by 1 partner to reduce connectivity by more than half in a network of 500 actors with 25% of the population connected. Thus an intervention with low efficacy at the individual level might still be effective at the population level. With the right data and the right models, a reliable intervention target can be identified and translated into an effective intervention program.
